# Topical allogeneic platelet-rich plasma treatment for a massive cutaneous lesion induced by disseminated intravascular coagulation in a toy breed dog

**DOI:** 10.1186/s13620-015-0032-7

**Published:** 2015-03-05

**Authors:** Tae-ho Chung, Dae-seung Baek, Namjung Kim, Jin-ho Park, Chul Park

**Affiliations:** Department of Animal Resources Science, Joongbu University, Chungnam, 312-702 Republic of Korea; Department of Veterinary Internal Medicine, BK21 Plus, Chonbuk National University, Jeonbuk, 561-756 Republic of Korea; National Academy of Agriculutral Science, Gyeonggi, 441-707 Republic of Korea

**Keywords:** Platelet-rich plasma, Disseminated intravascular coagulation, Cutaneous lesion, Toy breed dog, Topical treatment

## Abstract

A 2-year-old intact female miniature Pinscher weighing 1.7 kg with a body condition score of 2/5 was presented for acute vomiting, lethargy for 2 days, and large petechial skin lesions on the hip region including the tail. Acute pancreatitis was diagnosed by clinical signs, strong positive cPLI test, laboratory test and ultrasound appearance. While the clinical signs associated with acute pancreatitis had improved in 3–5 days, lesion of petechial appeared on the left hip region 7 days after the presentation, with a fast progression into a necrotic tissue along the left side hip. Allogenic platelet rich plasma (PRP) with Weibrich and Kleis method was administered to promote skin healing and regeneration. Gradual and complete improvement in the dog’s wound lesions was noted approximately 1 month after applying allogeneic topical PRP. In this case report, allogeneic PRP was applied to a large regional cutaneous defect caused by coagulopathy induced by acute pancreatitis. Topical application of PRP in this case was unique in that allogeneic PRP was used instead of autologous PRP for the first time in cutaneous soft-tissue wound management in the veterinary medical field.

## Background

Disseminated intravascular coagulopathy (DIC) is a complex set of events that arises when there is excess intravascular coagulation causing multiple-organ microthrombosis and paradoxical bleeding including the skin [1]. The bleeding is caused by either inactivation or excess consumption of platelets and/or clotting factors because of increased fibrinolysis and may have a gradual or acute onset. Once this process starts, changes in the patient’s status occur rapidly and require intense monitoring with subsequent changes in therapy as indicated [2]. The classic cutaneous manifestations of DIC present as skin wounds such as bruising, contusions, petechiae, purpura, acral cyanosis, hemorrhagic bullae, purpura fulminans, subcutaneous dissecting hematomata, or bleeding from wound or venipuncture sites [3].

Cutaneous wound healing requires a complex balance between matrix elements and growth factors and is dependent on multiple variables, including blood supply, defect size, tension, and mobility that affect the rates of healing and residual scarring [4]. Platelet rich plasma (PRP) not only enhances wound healing but also helps regenerate skin tissue [5]. Autologous PRP enhances wound healing and tissue regeneration and has been used as a source of growth factors in bone fracture treatment and in general wound healing for skin defects or dental mucosal wound care [6-8]. However, the current method of applying PRP for skin recovery used in veterinary studies is limited to autologous PRP applications [9,10].

This report describes a very small patient (toy breed, < 2 kg) with a large skin defect including the tail and subsequent use of topical allogeneic PRP as adjunct therapy.

## Case presentation

**Figure 1:**
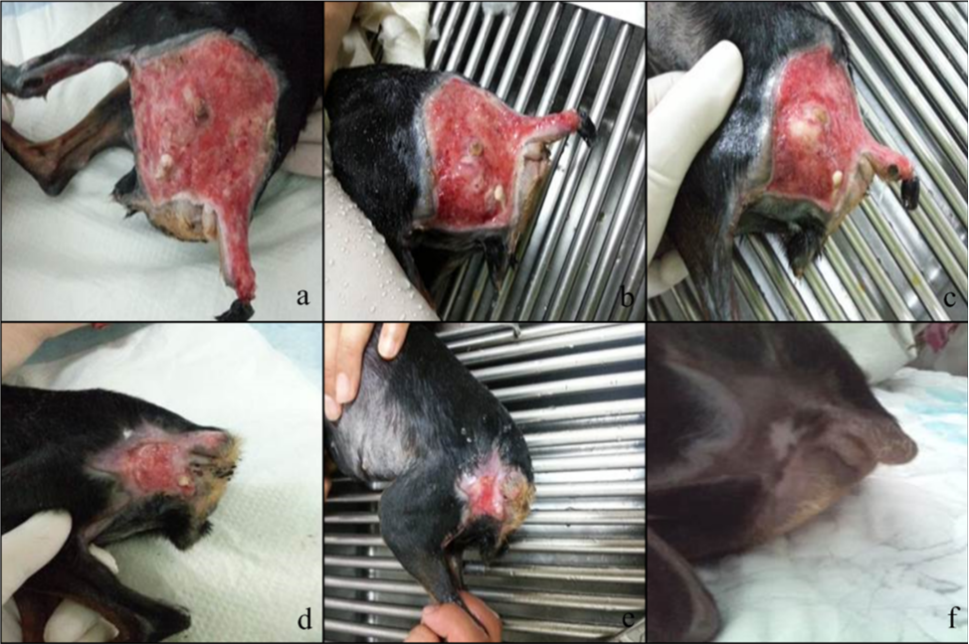
**Clinical improvement of lesions on (a) Day0, (b) Day2, (c) Day5, (d) Day13, (e) Day21 and (f) Day28 after topical PRP application.**

A 2-year-old intact female miniature Pinscher weighing 1.7 kg with a body condition score of 2 out of 5 was presented for acute vomiting, lethargy for 2 days, and large petechial skin lesions on the hip region including the tail. The dog was primarily diagnosed with acute pancreatitis based on clinical signs, strong positive cPLI test and abdominal ultrasound. The treatment was initiated mainly with fluid therapy (0.9% normal saline) and fresh frozen plasma (10 ml/kg) concurrently with other supportive treatment, including analgesics (Tramadol, 3 mg/kg, IV), antiemetics (Maropitant, 1 mg/kg, SQ), and H2-blockers (Ranitidine, 2 mg/kg, IV). While the clinical signs associated with acute pancreatitis had been improved in 3–5 days, lesion of petechial appeared on the left hip region 7 days after the presentation, with a fast progression into a necrotic tissue along the left side hip. The concurrent cutaneous manifestation of systemic DIC was recognized according to diagnostic references [11] by the acute pancreatitis diagnosis and laboratory results showing a low platelet count, prolonged blood clotting time, and increased fibrin degradation products such as D-dimer (Table 1). The skin lesions were diagnosed as small petechiea that appeared on the left stifle on the first day of presentation, with fast progression to necrotic tissue along the left side of hip within the 3 days. Initial treatments included debridement and warm saline washes with systemic cephalexin twice per day (Cephalexin; Ildong Pharm Co., Ltd. Seoul, South Korea) to protect against secondary infection. The lesions had not improved 5 days after the acute pancreatitis signs had improved (Figure 1a). Allogeneic PRP was added to the conventional treatments to promote skin healing and regeneration on day 5 because autologous PRP was impossible due to the patient’s low body mass (<2 kg, toy breed). The basic protocol to prepare the PRP was based on the Weibrich and Kleis method [12]. The PRP applied in this case was allogeneic PRP, instead of autologous PRP, in which the PRP blood source was from a donor dog (5-year-old intact healthy female beagle). Except for this difference, the other procedures for preparing the PRP were similar to those of the Weibrich and Kleis method, in which the donor’s blood (8 mL) was drawn into a citrate tube and centrifuged for 10 minutes at 629 × g to separate the blood into two basic components according to density (PRP in the top layer and red blood cells and white blood cells in the bottom layer). The PRP was carefully removed with a pipette and mixed with 200 μL of the lower layer, which contained large but more recently synthesized platelets. The mixture was centrifuged at 1,233 × g for 14 minutes to separate the platelet pellet in the supernatant layer. Centrifugation was repeated until the PRP mixture was obtained, including 0.6–0.7 mL of supernatant and a mean platelet concentration of 1.0–1.2 × 10 [6] platelets/μL. The PRP was mixed with an equal volume of sterile saline solution and 10% calcium chloride. The topical PRP gel (0.1 mL) was applied to the hip lesion, supplemented with erythropoietin (Epokine, Pharma CJ Cheiljedang, Icheon-si, Republic of Korea) and granulocyte colony-stimulating factor (Leukokine, Pharma CJ Cheiljedang, Icheon-si, Republic of Korea) for active hematopoiesis while continuing debridement and saline cleansing. Two days after applying the PRP, the inflammatory exudate decreased, and granulation tissue began to grow (Figure 1b). The lesions improved in color and mild regression in size was observed 5 days after applying the PRP (Figure 1c). The defect regressed significantly, almost by half in diameter, 13 days after applying the topical PRP (Figure 1d). Four weeks after the first presentation and 3 weeks after the first PRP application, the wound had significantly regenerated with healthy granulation tissue and was almost completely healed with epithelialization (Figure 1e). The skin lesions had almost completely regenerated 25 days after the allogeneic PRP application, which was 30 days after the first presentation of the skin lesion. A marked reduction in swelling, lack of crusts and erosion, and hair regrowth were observed (Figure 1f). This case trial has been carried out under the ethical guidelines of Chonbuk University IACUC.

**Table 1:** **Specific hematology, serum chemistry, and coagulation profile data of a dog with disseminated intravascular coagulation associated with pancreatitis**

**Hematology**	**Value**	**Reference interval**
WBC	19.41	6 ~ 12 (10x9/L)
WBC-Lymph(#)	1.1	1 ~ 3.6 (10x9/L)
WBC-Lymph(%)	5.8	0 ~ 100 (%)
WBC-Mono(#)	0.6	0 ~ 0.5 (10x9/L)
WBC-Mono(%)	3.2	0 ~ 100 (%)
WBC-Gran(#)	17.7	3 ~ 10 (10x9/L)
WBC-Gran(%)	91	0 ~ 100 (%)
WBC-Eos(%)	1.1	~ (%)
RBC	6.89	5.5 ~ 8.5 (10x12/L)
Hemoglobin[Hb]	15.69	15 ~ 20 (g/dL)
Hematocrit[Hct]	48.13	37 ~ 54 (%)
MCV	70	60 ~ 77 (fL)
MCH	22.77	17 ~ 23 (pg)
MCHC	32.61	31 ~ 36 (g/dL)
RDW-CV	14.43	14 ~ 17 (%)
Platelet	66	200 ~ 460 (10x9/L)
MPV	8.37	6.7 ~ 11.1 (fL)
**Serum chemistry**	**Value**	**Reference interval**
Albumin	2.2	2.2 ~ 3.9 (g/dL)
ALKP	159	23 ~ 212 (U/L)
ALT	61	10 ~ 100 (U/L)
Amylase	2500	500 ~ 1500 (U/L)
BUN	50	7 ~ 27 (mg/dL)
Calcium[Ca++]	8.4	7.9 ~ 12 (mg/dL)
Cholesterol-Total	200	110 ~ 320 (mg/dL)
Creatinine	0.4	0.5 ~ 1.8 (mg/dL)
Glucose	83	77 ~ 125 (mg/dL)
Phosphorus-Inorganic	3.4	2.5 ~ 6.8 (mg/dL)
Bilirubin-Total	1	0 ~ 0.9 (mg/dL)
Protein-Total	6.1	5.2 ~ 8.2 (g/dL)
**Coagulation panel**	**Value**	**Reference interval**
Whole blood APTT	99	60 ~ 93 (sec)
Whole blood PT	15.5	11 ~ 14 (sec)
D-dimer	0.7	0.1 ~ 0.5 (mg/dL)

## Conclusion

Studies on dogs and cats that have evaluated the ability of allogeneic PRP to heal cutaneous wound and regenerate tissue are extremely rare [5]. Conventional treatments for chronic wounds including topical steroids and antibiotics with changes of dressings and debridement sometimes do not effectively promote the wound-healing cascade as in the present case induced by DIC associated with acute pancreatitis. Although randomized controlled studies are extremely rare for DIC-induced skin defects associated with acute pancreatitis, PRP and platelet gels are increasingly being used for therapeutic tissue regeneration, as evidenced by several published clinical and experimental reports in human medicine.

PRP was applied to the non-healing wounds based on its potential to enhance healing of large wounds, and gradual and complete improvement in the patient’s tail lesion was noted approximately 1 month after applying the allogeneic topical PRP. Various hematological abnormalities including decreased serial hemoglobin or hematocrit values, coagulation factor abnormalities, leukocytosis, acute hemolytic anemia, thrombocytopenia, and thrombotic thrombocytopenic purpura or hemolytic uremic syndrome have been reported in patients with acute pancreatitis [13]. Similarly, abnormalities in blood coagulation factors consistent with DIC have also been detected in patients with pancreatitis.

PRP is a plasma fraction, in which platelet-derived growth factor and transforming growth factor-β are thought to be concentrated. PRP contains other autologous thrombocytic growth factors that might be promising for accelerating tissue regeneration. In the present case, the patient was too small with a poor body condition score of 2/5 and body fluid volume was extremely low due to dehydration to take blood to prepare autologous PRP. Gradual and complete improvement in the dog’s wound lesion was noted approximately 1 month after applying allogeneic topical PRP using a similar treatment period as that for autologous PRP [9]. Results showed an equally potent efficacy for wound healing as that of autologous PRP. This finding could open a new field of wound healing therapy, particularly in dogs with microangiopathy or coagulopathy and hormonal defects.

PRP is a cost-effective and readily available therapeutic blood derivative that is rich in growth factors and cytokines and increases tissue regeneration by affecting cell recruitment, proliferation, and differentiation. Applying allogeneic PRP topically to treat skin vascular defect-associated problems may be particularly effective because of the high leukocyte concentration, which results in local debridement and antibacterial activity without a rejection reaction in this case, particularly in toy breed dogs with a small body mass and fluid volume. Further studies with a larger number of samples in controlled clinical trials are required to elucidate a clear mechanism of action and identify possible adverse effects of using PRP for this application.

In conclusion, we demonstrated that topical treatment with allogeneic PRP is a safe adjunct therapy for skin wound healing. Allogeneic PRP may be particularly beneficial to manage large skin defects in toy breed dogs that are induced by DIC associated with acute pancreatitis.
